# Toward individualized medicine in stroke—The TiMeS project: Protocol of longitudinal, multi-modal, multi-domain study in stroke

**DOI:** 10.3389/fneur.2022.939640

**Published:** 2022-09-26

**Authors:** Lisa Fleury, Philipp J. Koch, Maximilian J. Wessel, Christophe Bonvin, Diego San Millan, Christophe Constantin, Philippe Vuadens, Jan Adolphsen, Andéol Cadic Melchior, Julia Brügger, Elena Beanato, Martino Ceroni, Pauline Menoud, Diego De Leon Rodriguez, Valérie Zufferey, Nathalie H. Meyer, Philip Egger, Sylvain Harquel, Traian Popa, Estelle Raffin, Gabriel Girard, Jean-Philippe Thiran, Claude Vaney, Vincent Alvarez, Jean-Luc Turlan, Andreas Mühl, Bertrand Léger, Takuya Morishita, Silvestro Micera, Olaf Blanke, Dimitri Van De Ville, Friedhelm C. Hummel

**Affiliations:** ^1^Defitech Chair of Clinical Neuroengineering, Neuro-X Institute (INX) and Brain Mind Institute (BMI), EPFL, Campus Biotech, Geneva, Switzerland; ^2^Defitech Chair of Clinical Neuroengineering, INX and BMI, EPFL Valais, Clinique Romande de Réadaptation, Sion, Switzerland; ^3^Department of Neurology, University of Lübeck, Lübeck, Germany; ^4^Department of Neurology, University Hospital and Julius-Maximilians-University, Wuerzburg, Germany; ^5^Hôpital du Valais, Sion, Switzerland; ^6^Clinique Romande de Réadaptation, Sion, Switzerland; ^7^Berner Klinik, Crans-Montana, Switzerland; ^8^Laboratory of Cognitive Neuroscience, INX and BMI, EPFL, Campus Biotech, Geneva, Switzerland; ^9^CIBM Center for Biomedical Imaging, Lausanne, Switzerland; ^10^Department of Radiology, Centre Hospitalier Universitaire Vaudois and University of Lausanne, Lausanne, Switzerland; ^11^Signal Processing Laboratory (LTS5), EPFL, Lausanne, Switzerland; ^12^The Biorobotics Institute and Department of Excellence in Robotics and AI, Scuola Superiore Sant'Anna, Pisa, Italy; ^13^Bertarelli Foundation Chair in Translational Neuroengineering, Centre for Neuroprosthetics and Institute of Bioengineering, School of Engineering, EPFL, Lausanne, Switzerland; ^14^Department of Clinical Neurosciences, University of Geneva (UNIGE), Geneva, Switzerland; ^15^Medical Image Processing Lab, Center for Neuroprosthetics, Institute of Bioengineering, EPFL, Lausanne, Switzerland; ^16^Department of Radiology and Medical Informatics, University of Geneva (UNIGE), Geneva, Switzerland; ^17^Clinical Neuroscience, Geneva University Hospital, Geneva, Switzerland

**Keywords:** stroke, precision medicine, transcranial magnetic stimulation, electroencephalography, neuroimaging, biomarkers, recovery, neurorehabilitation

## Abstract

Despite recent improvements, complete motor recovery occurs in <15% of stroke patients. To improve the therapeutic outcomes, there is a strong need to tailor treatments to each individual patient. However, there is a lack of knowledge concerning the precise neuronal mechanisms underlying the degree and course of motor recovery and its individual differences, especially in the view of brain network properties despite the fact that it became more and more clear that stroke is a network disorder. The TiMeS project is a longitudinal exploratory study aiming at characterizing stroke phenotypes of a large, representative stroke cohort through an extensive, multi-modal and multi-domain evaluation. The ultimate goal of the study is to identify prognostic biomarkers allowing to predict the individual degree and course of motor recovery and its underlying neuronal mechanisms paving the way for novel interventions and treatment stratification for the individual patients. A total of up to 100 patients will be assessed at 4 timepoints over the first year after the stroke: during the first (T1) and third (T2) week, then three (T3) and twelve (T4) months after stroke onset. To assess underlying mechanisms of recovery with a focus on network analyses and brain connectivity, we will apply synergistic state-of-the-art systems neuroscience methods including functional, diffusion, and structural magnetic resonance imaging (MRI), and electrophysiological evaluation based on transcranial magnetic stimulation (TMS) coupled with electroencephalography (EEG) and electromyography (EMG). In addition, an extensive, multi-domain neuropsychological evaluation will be performed at each timepoint, covering all sensorimotor and cognitive domains. This project will significantly add to the understanding of underlying mechanisms of motor recovery with a strong focus on the interactions between the motor and other cognitive domains and multimodal network analyses. The population-based, multi-dimensional dataset will serve as a basis to develop biomarkers to predict outcome and promote personalized stratification toward individually tailored treatment concepts using neuro-technologies, thus paving the way toward personalized precision medicine approaches in stroke rehabilitation.

## Introduction and rationale

With 80 million survivors in 2016, stroke is the second most common cause of acquired disability in the world (1, 2). This number is still increasing due to the population growth and aging (3). Better acute stroke management results in an improved stroke survival, but implies a higher prevalence of chronic stroke (2). Yet, complete motor recovery still occurs in <15% of patients (4). Moreover, although motor deficits are the most debilitating and investigated (5–7), patients also show consistent long-lasting cognitive deficits (8, 9), with a relevant proportion of patients having multiple domains affected. These long-term impairing behavioral deficits have a strong impact on patients' reintegration, on patients and their relatives' daily life, but also on socioeconomics and health care systems (10, 11). Therefore, the call for effective strategies of neurorehabilitation in order to maximize the rate of recovery is recognized as a priority to substantially reduce the burden of stroke survivors (2, 12). However, the heterogeneity in stroke outcome and in individual recovery potential is an important challenge to address, in order to provide optimal rehabilitative therapies. A crucial aspect to take up this challenge is to deepen our understanding of individual courses of recovery and the underlying neuronal mechanisms through the identification of associated biomarkers (13).

On the behavioral level, stroke is known to yield multiple deficits. The most reported and debilitating ones are the motor impairments, present in 50–80% of stroke survivors (7). In particular, damages to the upper extremity function are common and significantly impact the patients' capacity to retrieve independence, as well as to reintegrate to professional life (14, 15). Besides motor deficits, cognitive impairment is common in stroke survivors although initially less obvious: half of stroke survivors report difficulties in at least one cognitive domain, but this area is much less studied than the motor domain (8, 16). Cognitive impairment could be found in multiple domains most frequently in, e.g., executive functions, attentional functions or memory. Such deficits are significantly persistent after one to several years after the stroke (8, 17). Cognitive deficits also represent an obstacle for patients to go back into a normal daily life (10, 18, 19). Furthermore, these dysfunctions might strongly impact, slow or even prevent proper motor recovery and response to treatment (20). For example, it is known that executive functions, such as information processing and motor planning are essential in the processes of motor (re)learning (21), which is crucial in motor rehabilitation following stroke. However, despite few investigations of the relationships between these domains [e.g., 17, 22], research mainly focused so far on deficits in only one domain, e.g., motor (22), language (23) or attention (24) and neglected largely the interaction between them. Thus, there is a strong lack of knowledge about how deficits in different domains depend on and influence each other in regard of impairment, residual functions and the process of regaining lost functions after a stroke.

Recovery is often incomplete among stroke survivors, and the potential of restoring lost functions is crucially highly heterogeneous between patients (25, 26). For example, spontaneous natural recovery in motor domain occurs in roughly 2/3 of patients (13) who recover about ~70% in average of their maximum recovery potential given their initial impairment (27). In contrast, roughly 1/3 of patients presents altered or insufficient intrinsic plasticity after stroke leading to a poor natural recovery (13). Such heterogeneity has also been reported in other cognitive deficits e.g., neglect and aphasia (28). In addition, stroke survivors act highly heterogeneous in the view of the response toward specific treatment strategies, resulting in the distinction between responders and non-responders (29–31). For instance, patients with cortical lesions specifically demonstrated low responsiveness to repetitive Transcranial Magnetic Stimulation (rTMS) protocols (32, 33). Therefore, a key challenging aspect for enhancing neuro-rehabilitation efficacy might be to shed light on the heterogeneity of stroke patients and leverage this information to determine and predict the degree of impairment and potential for individual functional recovery (33, 34). This heterogeneity in stroke ranges from brain reorganization to behavioral outcomes and needs to be accounted for when planning rehabilitation strategies (31, 34).

The identification of specific individual patterns of recovery through a multi-domain perspective during the first weeks/months post-stroke, and crucially the uncovering of the underlying brain reorganization mechanisms would be a massive step toward the optimization of treatment strategies for each patient. However, there is a lack of understanding concerning the detailed neuronal mechanisms following a stroke lesion and during the course of recovery. Accumulating evidence suggests that stroke is not a focal disorder, but a brain network disorder (35, 36). In addition to local brain tissue damage, stroke also impacts the functioning of connected areas (close or remote from the lesion) as a result of alterations in brain networks (37). In addition, functional reorganization associated with recovery is also not restricted to a focal area. For instance, cortical plasticity associated with motor recovery is not restricted to the primary motor cortex (M1), but rather embraces the complete motor network, including primary and secondary motor cortical areas in both hemispheres, subcortical areas like the basal ganglia and the cerebellum (34, 37, 38). Factors such as lesion size and location [e.g., (39, 40)], as well as structural and functional prerequisites and dynamics (41) might relevantly influence recovery-associated plasticity processes in the brain leading to heterogeneous, widespread and time-dependent changes of brain reorganization and connectivity between patients. To improve rehabilitative strategies, it is therefore crucial to take this heterogeneity into account and understand how it relates to the pattern of network reorganization and the range of behavioral outcomes following a stroke.

On the basis of this reasoning, there is a strong need for an exact phenotyping of patients that would consider stroke heterogeneity in order to predict outcome and course of recovery and to further improve stroke recovery and treatment outcomes. Such challenge requires to gain a detailed and fundamental knowledge about the precise neuronal mechanisms associated with behavioral recovery, with a particular emphasis on brain networks changes. In addition, is essential to investigate the different domains impacted by the stroke instead of focusing on one behavioral outcome. As network and behavioral alterations following stroke are dynamic and not linear, a longitudinal investigation is of great importance. Such phenotyping will allow to distinguish distinct profiles of patients with associated dynamics of brain reorganization over the course of recovery. Enhancing the fundamental knowledge of stroke diversity through a multimodal and multidomain approach would serve as a basis to pave the way for personalized precision medicine in the field of stroke recovery to achieve maximal treatment effects.

To take up this challenge, the TiMeS project aims at characterizing in details phenotypes of stroke patients allowing to determine the individual course and degree of recovery following stroke and to identify relevant biomarkers associated with recovery. To that purpose, the goal is to collect a large multidimensional dataset that would be representative for the stroke population. Measurements will come from synergistic state-of-the-art systems neuroscience methods including magnetic resonance imaging (MRI), transcranial magnetic stimulation (TMS) coupled with electroencephalography (EEG), in a longitudinal assessment from acute to chronic stage during the first year after the stroke. As stroke is not a focal disorder, subsequent analyses will focus on networks properties within the whole brain and their changes over time, in combination with stroke behavioral outcomes with a focus on motor domain and further investigations of other neurocognitive domains. To provide detailed knowledge about the behavioral patterns and relationships between domains, the procedure will contain an extensive evaluation of behavioral outcomes in multiple domains, including a multi-domain cognitive assessment. The multidimensional dataset acquired through this research will enable to assess for the first time the complex interactions of structural and functional brain connectivity parameters within certain domain-specific networks as well as within the whole brain, and to associate them with stroke behavioral outcomes and functional recovery.

## Methods

### Study design

The present project is an on-going longitudinal observational study. We follow-up a total of up to 100 stroke patients at four timepoints over 1 year after the ictal event (T1: 1^*st*^ week, T2: 3 weeks, T3: 3 months, T4: 12 months) from the acute to the chronic phase of recovery. At each timepoint, we investigate the neural correlates of recovery and the underlying plasticity through a multi-modal and multi-domain set of evaluations including structural, diffusion, and functional neuroimaging (MRI), electrophysiology (resting-state EEG, and TMS coupled with EEG) and an extensive battery of tests assessing the multi-domain functional and behavioral outcomes of the patients.

### Objectives

The main goal of the study is to assess the inter-individual variance and different phenotypes of patients after a stroke (ischemic or hemorrhagic). The main goal is divided into two related objectives: (1) to evaluate the dynamics of neuro-imaging and neurophysiological factors associated with post-stroke course and degree of recovery with a focus on motor domain and structural and functional connectomics by means of multimodal MRI-based neuro-imaging and neurophysiological measures and, (2) to determine the interactions and the impact of different cognitive functions on residual motor functions and motor recovery after a stroke.

To complete these objectives, we apply a multimodal assessment of neuro-imaging and neurophysiological parameters to leverage the advantages of each method and account for their specific limitations to achieve a very detailed picture, especially in the view of the importance of network analyses. In addition, we use an extensive battery of behavioral tests to acquire detailed information concerning the patients' motor and cognitive profiles as well as their dynamics. The overall goal of this research will be to integrate and combine the multimodal data (i.e., neuroimaging, electrophysiology, and behavioral) together to obtain detailed and complete phenotypes of stroke patients. A list of all the measurements is provided in Table 1.

**Table 1:** List of measurements.

**Neuroimaging**
Diffusion-weighted imaging (DWI)
T1-weighted image
Multi-echo GRASE
BOLD functional MRI—Resting-state
GRE field mapping
Mp2rage
Susceptibility-weighted imaging
**Electrophysiology**
Resting-state EEG
TMS-EEG coupling
– Single pulse
– Double pulse (SICI)
**Behavior**
*Clinical evaluation*
NIHSS
**Motor functions**
Fugl-Meyer
Pinch&Grip
Medical Research Council muscle strength testing
Nine-hole peg test
Box and Blocks test
Purdue Pegboard Test
Action research arm test^*^
Modified Ashworth scale
2 min walk test^*^
10 m walk test^*^
Time up and go test^*^
Berg balance scale
**Sensory functions**
Rivermead assessment of sensory performance
**General cognitive screening**
Montreal cognitive assessment
**Attentional functions**
TAP—Phasic alert test
TAP—Divided attention test
D2-R
**Social cognition**
Geneva emotions recognition test—short^*^
**Executive functions**
Frontal assessment battery
Stroop Victoria
Bimanual coordination
Apraxia screen of test for upper-limb apraxia
CERAD constructional praxis
Color Trail Test
Bisiach anosognosia scale
Somatoparaphrenia test
5-points tests^*^
**Learning and memory**
Hopkins verbal learning test revised^*^ Doors test^*^
Digit span
Corsi-Kessels
**Perceptual function**
Overlapping figures test
Bisection line test
Bells cancellation test
**Questionnaires**
Stroke Impact Scale (SIS)
Hospital Anxiety and Depression Scale (HADS)
State/Trait Anxiety Inventory for adults (STAI)
Fear and stress scale
Medical outcome study short form 12
Modified reintegration to normal living index
Social comparison scale
Generalized self efficacy scale
Pittsburgh sleep quality index
Multidimensional fatigue inventory
Feeling of foreignness questionnaire
Neurobehavioral questionnaire
Barthel index
mRS modified ranking scale
FAC functional ambulation category
FIM functional independence
Edinburgh handedness inventory

* Means Tests not performed at T1.

### Primary outcome

As upper extremity function and impairment are the main reason for long-term disability and predictors of reintegration in normal life and functional independence after stroke, longitudinal recovery of the upper limb function and its underlying mechanisms are the primary interest of this study. Upper limb motor function includes multiple aspects, fine and gross dexterity, gross motor function, strength, spasticity, etc (42). These aspects are assessed longitudinally using the same set of reliable and validated clinical tests at each timepoint (see Appendix 1 for details). We are especially interested in how other cognitive domains and their alterations after a stroke impact on motor recovery.

### Secondary outcomes

Secondary outcomes are specific readouts based on the multi-domain cognitive evaluation and the multi-modal data from system neurosciences techniques, i.e., neuro-imaging and electrophysiological methods.

#### Magnetic resonance imaging (MRI)

Structural, diffusion-weighted and resting-state functional MRI are used to obtain individual structural and functional network properties to evaluate lesion-related neuronal alterations as well as their dynamics throughout the recovery phase, i.e., neuronal plasticity, reorganization and degeneration. Analyses will mainly focus on brain network alterations and changes over time through disconnectomics (43) and by applying computational approaches such as graph theory methods (44), e.g., the *Rich-Club* approach (39). In addition, integrated analyses of brain structure and function will be emphasized, e.g., by using the Structural Decoupling Index (SDI), a metric that allows to quantify the coupling strength between structure and function (45). MRI methods and sequences are detailed in Appendix 2.

#### Electrophysiological recordings

Functional measurements of the cortical excitability are provided by means of Transcranial Magnetic Stimulation (TMS). We use single pulses delivered to the primary motor cortex (M1) to generate motor evoked potential (MEPs) and to screen for cortico-spinal tract integrity. We also apply paired-pulses to assess the short-interval intracortical inhibition [SICI; (46)]. This is thought to reflect GABA_A_-mediated inhibition in the motor cortex (47). Electroencephalography (EEG) allows to assess the resting state brain connectivity (48). More importantly, in combination with TMS, EEG is used to assess interregional connectivity in the brain and to characterize the TMS-evoked potential and its evolution during the course of recovery. Therefore, TMS-EEG represents a unique method to study brain dynamics and their changes over time as it allows to record directly and non-invasively various neurophysiological processes across motor and non-motor areas e.g., cortical responsiveness, cortico-cortical interactions, local excitation and inhibition, oscillatory activity etc [see Tremblay et al. (49) for a recent review]. Electrophysiological methods are detailed in Appendix 3.

#### Behavioral outcomes

To assess precisely the motor and cognitive profiles of the patients, an extensive battery of 40 tests is performed at each timepoint by a trained neuropsychologist. The battery covers sensory-motor domains as well as each neuro-cognitive domain as defined in the DSM-V, i.e., executive functions, language, complex attention, learning and memory, social cognition, perceptual-motor domains (50). Multiple questionnaires complete this battery to evaluate additional aspects such as fatigue, mood, functional independence and recovery. See Appendix 1 for details.

### Study organization

#### Ethical considerations

The study was designed and is conducted according to the guidelines of the Declaration of Helsinki. All the procedures were approved by the cantonal ethics committee (Project ID 2018-01355, CER-VD, Vaud, Switzerland).

#### Eligibility

We address stroke patients presenting some upper limb motor impairment in the acute stage. In order to obtain a heterogeneous cohort, we screen patients with first-ever as well as recurrent stroke, either ischemic or hemorrhagic. Detailed inclusion and exclusion criteria are following:

- Inclusion criteria.
º Age > 18 years old.
º First-ever or recurrent stroke.
º Ischemic or hemorrhagic stroke.
º Stroke incident <7 days at consent.
º Motor impairment in the acute stage, objectified by a clinical assessment.
º Absence of contraindication for Non-Invasive Brain Stimulation (NIBS) and MRI.

- Exclusion criteria.
º Severe neuropsychiatric (e.g., major depression, severe dementia) or medical disease.
º Not able to consent.
º Severe sensory or cognitive impairment or musculoskeletal dysfuntions prohibiting to understand instructions or the perform the experimental tasks.
º Implanted medical electronic devices or ferromagnetic metal implants, which are not MRI and TMS compatible.
º History of seizures.
º Medication that significantly interacts with NIBS being benzodiazepines, tricyclic antidepressant and antipsychotics.
º Pregnancy.
º Regular use of narcotic drugs.
º Request of not being informed in case of incidental findings.

#### Recruitment and screening

Stroke patients are recruited at the stroke unit of the *Hôpital du Valais* (HVS), Sion, Switzerland. The member of staff in charge of the recruitment daily checks the list of new entries at the hospital. When a patient is eligible (see Inclusion and Exclusion criteria), the medical staff is consulted, and a first screening visit is organized with the patient. The study is presented in details to the patient, and eligibility is further evaluated. Patients are provided with 24-h for reflection in regard of participation before signing the consent to participate. If the patient consents, the first visit (T1) is organized during the first week after the stroke, while the patient is most of the time still hospitalized. The procedures are performed in accordance with the ethical approval.

#### Data acquisition and follow-up

The 1^*st*^ behavioral evaluation and the MRI acquisition are performed at the HVS. The electrophysiological measurements are performed in the laboratory, located in the *Clinique Romande de Réadaptation (CRR)* physically connected to the HVS. The total measurement time is of around 10 h, distributed in several sessions.

The patients enrolled in the study are then transferred for rehabilitation from the HVS to one of the two rehabilitation clinics collaborating within the present study, that is the CRR and the Berner Klinik (BK; Crans-Montana, Valais, Switzerland) or to home. The 3 weeks (T2) behavioral evaluation is performed during the in-patient stay, or in the laboratory if the patient was sent back home after the acute phase. For the 3 months (T3) and 12 months follow-ups (T4), patients are invited to our laboratory on the HVS/CRR campus for behavioral, MRI and electrophysiological recordings. We will analyze the different behavioral domains individually but we also aim to integrate the multimodal data together in statistical models and computational approaches, in order to determine interactions between the different parameters.

### Data management, planned analyses and statistical considerations

Based on previous comparable project [e.g., Grefkes and Ward (51); *N* = 132 patients] and given the estimated feasibility of our extensive multi-modal and multi-domain evaluations, we aimed to recruit up to 100 patients, with a recruitment rate of up to 40 patients a year. The minimal number of patients to be recruited is 80 at T1. So far, we recruited 86 patients in the acute phase. As the study is mainly explorative in its nature, we did not conduct a classical power calculation. The multi-modal aspect of the project includes a very large number of behavioral outcomes as well as numerous neuroimaging and electrophysiological variables. Because of this, the high risk of Type 1 error due to the use of a large number of statistical tests might be carefully considered through appropriate corrections and the reduction of data dimensionality. We report here the strategies planned to analyses the multi-dimensional data obtained

#### Behavioral planned analyses

The purpose of using an in-detailed set of behavioral assessments is to get a complete picture of behavioral functions after stroke and their dynamics in all domains, while avoiding the unique use of component scores to describe behavior. However, our extensive assessment battery entails a very large number of variables, which could lead to some redundancy between tests. Therefore, the first planned analyses regarding the behavioral dataset will be mainly descriptive to better understand the dispersion of performances and inter-individual variability for each test within each behavioral domain. Demographic and clinical information (i.e., sex, age, level of education, side and type of lesion, etc) will be systematically added in analyses as covariates. A second step will be to do a first investigation of relationships between variables using correlation matrices both within and between domains. These steps will enable a qualitative selection of variables to restrain the number of informative features for further analyses.

Clustering (e.g., *k-means*) analyses will then be used to investigate the emergence of different behavioral profiles within the cohort based on specific subset of variables, as well as their dynamics across time. These variables of interest will be selected based on previous exploratory analyses and/or on specific hypotheses from the previous literature (e.g., the existence of a strong relationship between motor impairment and attention; 70).

Further analyses using mixed models and multivariate linear regressions will enable to investigate early behavioral cognitive predictors of the post-stroke motor recovery, i.e., whether specific cognitive performances in the acute phase predict the course of motor recovery.

Finally, dimensionality-reduction methods such as principal component analysis and nonnegative matrix factorization (52) will be used to transform the large number of variables into smaller number of component scores specific to each behavioral domain. Therefore, the investigations of relationships between behavioral outcomes and neuro-imaging/electrophysiological features will be conducted using qualitatively selected variables from the battery and/or using component scores.

#### Neuroimaging planned analyses

Voxel lesion symptom mapping will be used to investigate the relationships between behavioral outcomes and lesion sites. Neuroimaging analyses will then focus on brain connectivity features through structural connectomics, which rely on models of white matter tractography computed from diffusion-weighted imaging, as well as resting state functional connectomics. Following methods will be applied to reduce dimensionality of the dataset with high complexity. For each patient, we will compute a total connectome and an unaffected connectome, in order to incorporate the paths that have been disrupted by the lesion, together with respective scales of network science like e.g., global efficiency, a metric reflecting the functional integration within networks (53, 54). Principal component analyses will be performed, as it has been shown to reduce dimensionality whilst remaining high subject specificity when being used on whole brain connectomes (55). Further individual structural and functional connectivity of specific functional networks like sensorimotor and attention will be reconstructed. These features will be then related to post-stroke impairments, with a first focus on the sensorimotor and attentional domains as the networks underlying those functions are known to be respectively heavily localized vs. more global (56).

Besides, integrated analyses of brain structure and functions will be performed using the Structural Decoupling Index [SDI; (39)] to quantify the coupling strength between structure and function and how this could be impacted by stroke within the different brain networks. Using individual and functional structural connectome, SDI will be computed for each patient, each timepoint and within each Yeo brain network (57). Partial Least Square Correlations [PLSC; (58)] will be then used to identify multivariate correlation patterns between patient-specific nodal SDI measures and behavioral component scores for each domain (59).

#### Electrophysiological planned analyses

We expect a large heterogeneity of TMS evoked activity patterns within the brain among stroke patients and between single-pulse and double-pulse paradigms. In addition, the purpose is to compute TMS-EEG readouts that can be individualized in order to phenotype patients. Therefore, we plan to employ complex analytic measures beyond the classical use of grand average event-related potential (40, 60, 61).

We will compute the local mean field power (LMFP) which reflects the cortical reactivity (62) and the number of deflections of the local TMS evoked potential, which reflects the complexity of the signal (61). Besides, Regression Quality Scores (RQS) will be used to assess the cortical response stability within one given timepoint (paired RQS) and level of similarity of cortical responses dynamics between two given timepoints (unpaired RQS) (40). In the frequency domain, the exploration of brain oscillations, either at rest or triggered by TMS, will be of interest (63). Finally, the impact of stroke on functional and effective connectivity will also be explored in the source space (64). Those precise readouts will be then correlated with specific motor and cognitive scores as well as their evolutions across the different timepoints.

#### Statistical considerations

Therefore, we will use a broad spectrum of statistical tools designed for high-dimensional datasets, like mixed-effects models but also Bayesian statistics (Bayesian equivalent of ANCOVAs and Kendall correlations) by using Bayes Factor as the indicator of statistical evidence (65). All the statistics will be performed using either R software (2017, R Core Team, Vienna, https://www.Rproject.org), the SPSS software (2017, IBM SPSS Statistics for Windows, IBM Corp, Armonk, New York), the JASP software, Matlab (v2020b, Mathworks, The MathWorks, Massachusetts, http://www.mathworks.ch) and/or Python (2009, CreateSpace, Scotts Valley, California).

Implementing strategies to reduce the dimensionality of the data while keeping the richness of the planned multi-modal together with the planification of a priori specific analyses to conduct enable to anticipate issues related to multiple comparisons. These will be anyhow systematically and rigorously assessed in the end using state-of-the art statistical methodologies of corrections. For instance, *p*-values will be systematically corrected within frequentist framework (e.g., using Bonferroni's corrections) while Bayesian Factors will be systematically reported for Bayesian analyses. Nevertheless, it is important to mention that due to the exploratory nature of the project, the initially planned specific analyses will certainly drive further complementary analysis based on specific hypotheses arising from the first insights. Overall, the ultimate objective will be to apply machine learning tools as classifiers, supervised, unsupervised and deep learning algorithms as they provide the opportunity to derive insights from imaging and electrophysiological data coupled with behavior to produce predictive models and to discovering phenotypes of patients (66, 67).

## Discussion

As depicted in the introduction, stroke results in multi-domain behavioral deficits in survivors. Although motor deficits (in particular in the upper extremity) are the most impairing, the prevalence of cognitive deficits is also highly important and concerns multiple domains. In addition, they were demonstrated to likely impact the functional recovery and the reintegration in life following stroke, as well as the outcomes of motor rehabilitation (20). Yet, little attention has been paid so far to how cognitive and motor domains are related and influence each other following stroke. Consequently, there is a lack of detailed phenotyping of behavioral outcomes and their evolution though it would be of high interest to improve rehabilitation tailoring (68, 69).

Several studies have investigated the relationships between cognitive and motor outcomes (17, 68, 70–73) and showed that cognitive impairments were common even in patients with mild strokes, and that relationships exist between motor and cognitive domains. This highlights the relevance of such multi-domain approaches, emphasizing that motricity and cognition should not be investigated separately. For instance, Einstad et al. (64) have recently demonstrated that poor motor performances are associated with impaired global cognition scores and executive dysfunctions. However, such studies made use of a limited battery of tests and/or focused on one particular timeframe during stroke recovery without any longitudinal assessment (i.e., acute, sub-acute, chronic). Ramsey et al. (17) employed a battery of motor and cognitive tests to evaluate the patients over the course of recovery during the first year; at 1–2 weeks, 3 months and 1 year after the stroke. They reported that across multiple domains, sub-acute scores were strong predictors of the performance in the chronic stage and that the magnitude and time course of recovery were comparable between cognitive and motor domains. Specific behavioral clusters were identified (e.g., a strong relationship between motor impairment and attention) and shown as being stable over the three timepoints. In addition, the authors described relationships of interest between domains over the course of recovery (e.g., language deficits influenced the recovery of verbal memory). Interestingly, the authors studied how lesion topography could explain behavior, as it was done in another study from the same group (68) and pointed out that white matter damage could be a key feature in explaining behavioral recovery. Other studies from the same cohort independently investigated the relationships between resting-state fMRI data and behavior by showing that altered functional connectivity correlated with behavioral deficits in the motor and attention domains (74) and in hemi-spatial neglect (75). In addition, the authors demonstrated that memory deficits are better predicted by functional connectivity than by lesion topography while the motor and visual deficits might be better predicted by lesion location than functional connectivity (76). Altogether, these studies emphasized the importance of multi-domain behavioral assessments and the interest of investigating brain-behavior relationships both through structural and functional measures as they provide complementary insights. However, patients enrolled in this cohort were substantially younger than the natural population of stroke survivors [average age 54 ± 11 years old, range 19–83, benchmark 69.2 years in 2005 Greater Cincinnati/Northern Kentucky cohort; (77)] and executive functions were not assessed in the battery. Plus, the authors focused on one modality (MRI) to assess brain features which provides rich but limited insights about the neuronal mechanisms underlying post-stroke recovery. To date, no study provided any extensive behavioral evaluation (with an approach centered on the individuals rather than the whole cohort) and during the course of recovery following stroke while combining data with multimodal assessments of brain network plasticity.

A common factor in many of these studies is the interplay between structural and functional connectivity. Structure influences function in the obvious way, while function influences structure in the long term. However, there is strong evidence that the strength of the link between structure and function is domain-dependent. The findings of Siegel, Ramsey et al. (17) suggest that function is tightly coupled to structure in the motor and visual domains, while the two are more decoupled for “higher order” domains such as memory. These findings have been echoed in Preti and Van De Ville's work (2019), which found that brain regions responsible for “low level sensory function” tend to exhibit strong structural-functional coupling, and vice versa.

The present study aspires to bolster our understanding of mechanics underlying multiple-domain deficits by providing a multi-modal and multi-domain evaluation of stroke patients longitudinally during the first year after the stroke. This research intends to investigate the different behavioral profiles and their dynamics in stroke patients, not only looking at the motor domain but undergoing a holistic approach coupled with neuro-imaging and electrophysiological parameters. Therefore, the originality of the project lies in the multiplicity of the approaches undertaken that will allow a very detailed picture of the recovery and the reorganization in the brain following stroke. Structural, diffusion-weighted and functional MRI will provide the opportunity to study network dysfunctions as well as the complex interactions between brain function and structure. In addition, simultaneous EEG recording during TMS is a promising approach that will enable to explore brain connectivity and recovery pattern for functional networks after stroke by providing a direct measure of the cortical activity induced by TMS. By combining modalities with different advantages (such as either excellent spatial or temporal resolution, structural vs. functional information) and by following patients along the first year post-stroke, we will provide a complete dataset allowing to integrate multimodal information in statistical and computational models. The overall goal is to determine interactions between the different parameters as well as factors usable as biomarkers for phenotyping patients in regard of the course and the degree of recovery.

Identifying such biomarkers might help (1) to predict the course of recovery, i.e., to early detect patients that will spontaneously recover and those who will not and, consequently, (2) to personalize the therapeutic strategies in order to meet the individual needs of each patient and to maximize the treatment benefits. Therefore, this work will serve as a basis for improving existing treatments or developing novel and innovative ones tailored to the individual patients' characteristics by providing a better understanding of neural mechanisms underlying successful recovery. For instance, NIBS are neuro-technologies that are more and more used in stroke rehabilitation to promote motor recovery (31, 78, 79) due to their noninvasiveness, relatively low cost and limited side effects. However, there is a high heterogeneity in the outcomes (29, 30, 67, 80, 81): effects of NIBS are still limited, which can be partly explained by the use of non-personalized approaches (31, 82). Some biomarkers have already been identified to stratify patients in order to assess the individual recovery potential, for instance the cortico-spinal tract integrity as measured by presence or absence of MEP (83). However there is still a lack of fundamental knowledge on the topic especially considering the longitudinal changes in brain dynamics following stroke (22). The detailed phenotyping based on the dataset from the present study might further help to provide extra layers of stratifications allowing more precise predictions about treatment outcomes in order to reduce the number of non-responders (67). Therefore, some potential perspectives are to further design interventional studies to analyze the efficacy of neurotechnologies-based treatment personalized thanks to clustering and stratifying algorithms arising from this research.

## Challenges and limitations

Since this work involves plural and extensive multi-modal assessments, it is worthwhile to emphasize that the patients need to be physically and mentally capable of undergoing such multiple recordings. Plus, as the patients need to understand what the project entails, severe language deficits prevent possible participants to be enrolled because they do not have the ability to consent while being transparently informed. Furthermore, the presence of TMS recordings is associated with a consistent list of exclusion criteria related to medication, epilepsy or implants (metallic or electronic) that could interact with the stimulation. These aspects might cause a bias in the recruitment of patients that we need to consider when interpreting the results. Plus, we decided to include both first-ever and recurrent stroke patients to obtain a cohort that is representative of the stroke population while most of the performed neuroimaging studies excluded recurrent stroke, thus limiting the acquired understanding to this group of patients. Although we hypothesize that connectomics will allow to gain interesting information in this regard, we will carefully take this aspect in caution in second-level analyses and interpretations, as some residual impairments may be related to previous lesions in patients with a recurrent stroke. On the other hand, the presence of an upper-limb motor deficits is an inclusion criterion as the initial purpose of the project is to investigate post-stroke motor recovery, and factors that impact on residual motor function and degree and course of motor recovery. The investigation of interactions between cognitive and motor domains might be biased as we do not explore these interactions in patients with cognitive deficits but no motor impairment. We nevertheless aim to recruit a cohort as heterogeneous as possible to cluster patients and identify specific patterns of recovery and brain reorganization. Plus, it is crucial to point out that motor deficits are very common in the general stroke population [~80–85% of patients; (17, 83)], indicating that conclusions from the present study will be within the framework of a clinically relevant subgroup of patients population. In addition, we still expect to observe varying degrees of motor impairment, from very slight to severe, which can reduce the risk of biased interpretations concerning the relationships between cognitive and motor domains.

Other challenges relate to the longitudinal aspect of the project. First, the four timepoints might be insufficient to capture some fine temporal changes in brain connectivity and behavior. However, the extensive and multi-modal nature of the study requires many resources and represent a large amount of time testing per patient. Although the current protocol is feasible thanks to the physical location of the laboratory close to the hospital and the rehabilitation clinics (for details, see Appendix 4), adding more timepoints would have seemed unrealistic. Still, the chosen temporal resolution will allow to address the recovery process relatively to the main key time frames post-stroke, i.e., acute, subacute, early chronic and late chronic. Second, drop-outs are common for this type of study and we expect some missing data points. Specifically, there is a higher chance of loss for the most impaired patients as the drop-outs are likely to be related to bad medical condition for example or a lack of motivation. This needs to be carefully considered in the choice of the statistical tools and in the interpretations of results. Still, efforts will be maintained to avoid drop-outs, e.g., by maintaining contact with the patients between timepoints and by facilitating their visits during the follow-up (see Appendix 4).

Finally, it is crucial to emphasize the exploratory aspect of the study. Systems neuroscience methods in humans such as MRI-, EEG- or TMS-based recordings represent associative approaches with limitations to unquestionably proof causality. For example, the post-stroke changes in brain connectivity observed through neural measurements could be due to effects of the lesion which are not related to recovery. Alternatively, they can be related to reactive changes associated with recovery but that do not directly cause it. To better address these aspects, our analyses are not limited to the lesion or specific areas and/or tracts, but rather focus on connectomics which provide rich information in the understanding of stroke-induced deficits, recovery process and prediction [e.g., (41, 83–85). Thus, the proposed multi-modal approach will allow to better capture brain reorganization features related to the stroke.

## Summary and conclusion

A better understanding of the neuronal mechanisms associated with recovery-related plasticity and reorganization of the brain networks after a stroke is needed to enhance the understanding of the recovery process, and to predict the outcome and course of recovery. This knowledge will enable to develop and apply interventional strategies in a personalized way to enhance the effects of the treatments for each individual patient. The TiMeS project is a longitudinal, multimodal, and multidomain study of a large, representative cohort of patients during the first year after the stroke, including structural and functional neuro-imaging, electrophysiological and extensive behavioral evaluations. This exploratory research will provide the opportunity to integrate and combine multidimensional data from neuroscience systems methods together with detailed behavioral outcomes to identify specific biomarkers of recovery. This phenotyping will serve as a basis to tailor current rehabilitation strategies according to each patient's individual needs and to develop innovative personalized neuro-technologies based treatment like NIBS, beyond a one-fits-all approach. Overall, the knowledge gained from this study will pave the way for establishing a close link between basic neuroscience and the development of novel treatments into clinical routine toward precision medicine in stroke, which is highly promising to reduce the burden of the disease.

## Ethics statement

The study involving humans participants was reviewed and approved by Cantonal Ethics Committee Vaud, Switzerland (project number 2018–01355). The patients/participants provided their written informed consent to participate in this study.

## Author contributions

PJK, MJW, TM, and FCH researched literature and conceived the study, and were involved in ethical procedures. LF is involved in patient recruitment, data acquisition, data analysis, researched literature, and wrote the first draft of the paper. CB, DSM, CC, PV, JA, CV, VA, JT, ACM, and BL are clinical partners and were involved in the conception, and recruitment of the study. ACM, JB, EB, MC, PM, DD, VZ, and NHM are involved in patient recruitment, data acquisition, and data analysis. ACM and JB draft the methodological appendices (electrophysiological and neuroimaging parts). SH and GG substantively revised it. All authors reviewed and edited the manuscript and approved the final version of the manuscript.

## Funding

This work was supported by ‘Personalized Health and Related Technologies' (PHRT-205) of the ETH Domain, the Defitech Foundation (Strike-the-Stroke project, Morges, Switzerland) and the Wyss Center for Bio and Neuroengineering (Geneva, CH). Open access funding is provided by École Polytechnique Fédérale de Lausanne.

## Conflict of interest

The authors declare that the research was conducted in the absence of any commercial or financial relationships that could be construed as a potential conflict of interest.

## Publisher's note

All claims expressed in this article are solely those of the authors and do not necessarily represent those of their affiliated organizations, or those of the publisher, the editors and the reviewers. Any product that may be evaluated in this article, or claim that may be made by its manufacturer, is not guaranteed or endorsed by the publisher.

## Supplementary material

Datasheet 1 Neuropsychological evaluation details.

Datasheet 2 Neuroimaging recordings details.

Datasheet 3 Electrophysiological recordings details.

Datasheet 4 Study organization.
