# Supplementary material for: Toward individualized medicine in stroke—The TiMeS project: Protocol of longitudinal, multi-modal, multi-domain study in stroke
Source: Front Neurol. 2022 Sep 26;13:939640. doi: 10.3389/fneur.2022.939640 (PMC9549862; doi:10.3389/fneur.2022.939640)
Supplement: Datasheet 4 — Study organization. [file Data_Sheet_4.docx]

**Supplement 4 – Study organization**

The proposed multi-modal and longitudinal protocol entails a large and time-consuming number of recordings (see Figure 1). Although this project is highly challenging especially with severely impaired patients, the protocol is feasible thanks to several aspects. The MRI facilities as well as our laboratory are in close vicinity of the acute hospital (HVS) and the rehabilitation clinic (CRR), see figure 2. This facilitates the recruitment, but also the evaluations and recordings, especially during the acute and subacute phase (T1 and T2). In addition, we established an excellent relationship with the medical staff of the hospitals which facilitates the integration of the testing into the clinical work and rehabilitation schedule. We also have materials for neuropsychological testing in the laboratory, at the hospital, and at both clinics and specific rooms dedicated to test the patients directly in the hospital/clinic, while keeping a high reproducibility. Another aspect is that we have good access to transportations facilities through a partnership with local transportations companies and associations to offer the patient the possibility to easily come to the laboratory. As we recruit from the local regional hospital, most patients live in a close vicinity to the laboratory. Noticeably, a member of the staff entirely work to the whole organization of patients visits, including contact with patients or relatives, medical staff, and transportations facilities.

In addition, we established a hierarchy in the different modalities of assessments (e.g., the order of the MRI sequences performed, importance of the behavioral scales). Therefore, we could adjust to the patients ‘needs and schedules optimally. Concerning the behavioral evaluations for example, two sessions are initially planned but can be divided into more if the state or the rehabilitation schedule of the patient require to do so.

All these organizational aspects allow to facilitate the recruitment and the longitudinal follow-up of the patients with flexibility in regard to their personal schedule and state.


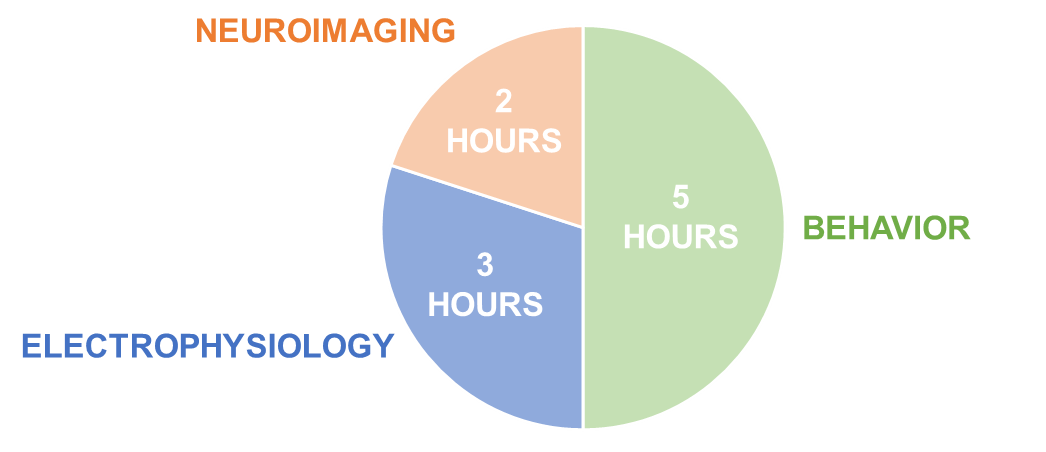


Figure 1 - Hours of testing per timepoint


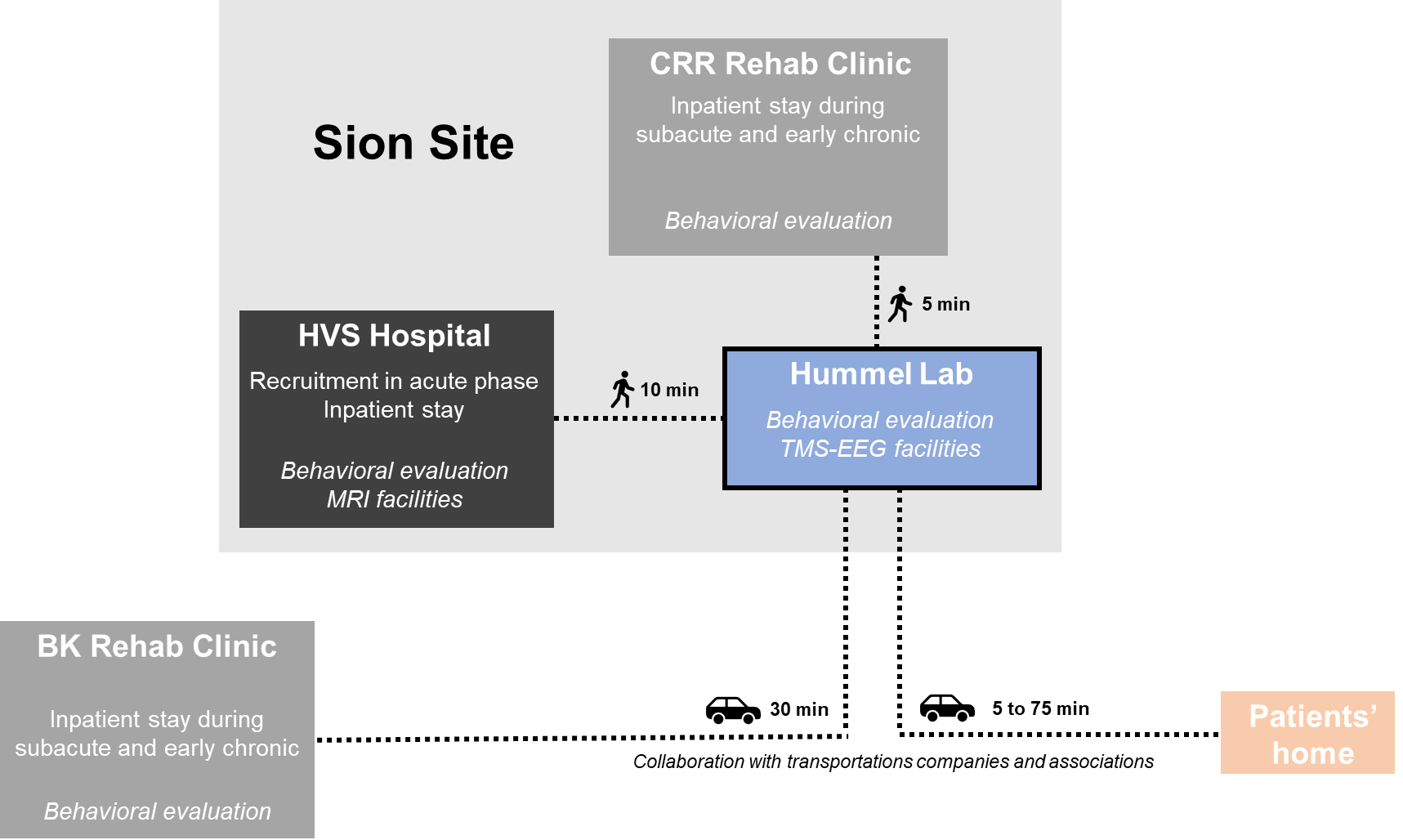


Figure 2 - Organization between the different structures and sites
